# Angiotensin II Receptor‐Associated Protein (AGTRAP) Enhances Glioma Cell Survival Through the IL‐6/JAK2/STAT3 Pathway and Correlates With an Immunosuppressive Microenvironment

**DOI:** 10.1002/cns.70796

**Published:** 2026-02-13

**Authors:** Siyu Chen, Yuntao Li, Xiaohu Nie, Yonggang Zhang, Qianxue Chen, Xiaoxing Xiong, Zhongzhou Su, Sheng Qiu

**Affiliations:** ^1^ Department of Neurosurgery, Huzhou Central Hospital Huzhou University Central Hospital Huzhou China; ^2^ Huzhou Central Hospital The Fifth School of Clinical Medicine of Zhejiang Chinese Medical University Huzhou China; ^3^ Huzhou Key Laboratory of Basic Research and Clinical Translation for Neuromodulation Huzhou China

**Keywords:** AGTRAP, apoptosis, glioma, IL‐6/JAK2/STAT3 pathway, macrophage, proliferation, tumor microenvironment

## Abstract

**Aim:**

There is an urgent need for actionable therapeutic targets for glioma. Angiotensin II receptor‐associated protein (AGTRAP) is upregulated in glioma, but its functional role and downstream programs remain insufficiently defined. This study aimed to clarify the clinical relevance, biological function, and mechanism of AGTRAP in glioma.

**Methods:**

AGTRAP expression and clinicomolecular associations were analyzed across public glioma cohorts. Loss‐of‐function studies were performed in glioma cells (A172 and U251), followed by proliferation and apoptosis assays. Recombinant IL‐6 was used for rescue experiments. An orthotopic xenograft model was used to evaluate tumor growth in vivo.

**Results:**

AGTRAP expression is significantly elevated in gliomas versus normal brain tissues and correlates with tumor grade, age, 1p/19q co‐deletion, and IDH mutations. High AGTRAP expression predicted poorer survival. AGTRAP knockdown suppressed proliferation, increased apoptosis, reduced IL‐6 mRNA and protein levels, and attenuated JAK2/STAT3 activation. Recombinant IL‐6 partially restored JAK2/STAT3 signaling and mitigated the growth‐inhibitory phenotype caused by AGTRAP silencing. In vivo, AGTRAP knockdown reduced tumor burden. Transcriptome‐based analyses showed that AGTRAP expression was associated with a myeloid/macrophage‐enriched microenvironment, and exploratory analyses suggested cross‐tumor associations between AGTRAP expression and checkpoint blockade outcomes.

**Conclusion:**

AGTRAP supports glioma cell survival by engaging an IL‐6–linked JAK2/STAT3 program and is associated with a macrophage‐rich, inflammatory tumor microenvironment. These findings suggest that AGTRAP may serve as a candidate intervention target for gliomas.

## Graphical abstract

AGTRAP is upregulated in gliomas and is associated with adverse clinicomolecular features and poor prognosis. Mechanistically, AGTRAP maintains an IL‐6/JAK2/STAT3 survival axis that promotes proliferation and limits apoptosis, and IL‐6 add‐back partially restores JAK2/STAT3 signaling after AGTRAP silencing. AGTRAP‐high tumors also display microglia/macrophage‐enriched immune signatures.

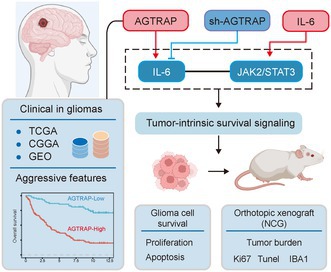

## 1 Introduction

Gliomas are the most prevalent primary tumors of the central nervous system (CNS) and remain a major cause of cancer‐related morbidity and mortality. The 2021 WHO Classification of Tumors of the Central Nervous System emphasizes integrated histo‐molecular diagnosis and highlights that higher‐grade gliomas are consistently associated with worse outcomes [1, 2]. Among these entities, glioblastoma (GBM) represents the most aggressive form. Despite maximal safe resection followed by radiotherapy with concomitant and adjuvant temozolomide, survival remains limited, with median overall survival on the order of 14–15 months in landmark clinical studies and contemporary practice [3, 4, 5]. Accordingly, defining actionable molecular vulnerabilities and mechanism‐based therapeutic strategies remains an urgent unmet need.

Angiotensin II receptor‐associated protein (AGTRAP) is classically enriched in cardiovascular and renal tissues, where it interacts with angiotensin II type 1 receptor (AT1R/AGTR1) and regulates receptor trafficking and internalization, thereby constraining pathological Ang II–AT1R signaling in hypertension‐associated inflammation and organ injury [6, 7]. Emerging evidence, however, suggests that AGTRAP functions are context‐dependent and can extend beyond canonical AT1R modulation. For example, AGTRAP has been reported to interact with transferrin receptor 1 (TfR1) and facilitate its internalization, providing a precedent for AT1R‐independent interaction networks connected to cellular stress biology [8]. In glioma, AGTRAP is reported to be upregulated and associated with prognosis in transcriptome cohorts [9, 10]. However, the crucial question remains whether AGTRAP is simply a correlative biomarker or a tumor cell–intrinsic driver of malignant phenotypes.

In this study, we integrate multi‐cohort bioinformatics analyses with experimental validation to characterize AGTRAP expression patterns and clinical relevance in glioma, establish its tumor cell–intrinsic impact on proliferation and apoptosis, and delineate an IL‐6–linked JAK2/STAT3 program downstream of AGTRAP. We further present immune/stromal and therapeutic‐sensitivity analyses as hypothesis‐generating observations to motivate future glioma‐centered validation.

## 2 Methods

### 2.1 Acquisition and Preprocessing of Data

All glioma cohorts were obtained from public databases, TCGA, UCSC, CGGA and GEO were processed and normalized, while microarray data underwent background effect correction using “Affy” package [11, 12]. Batch effects across all cohorts were adjusted using the “sva” package [13]. Genomic mutation data were retrieved from the UCSC database and analyzed using the “maftools” package. A pan‐cancer analysis of TCGA data was conducted using the TIMER website [14]. Besides, glioma grading information used in TCGA and CGGA analyses is based on the pre‐CNS5 (WHO 2016) criteria.

### 2.2 Single‐Cell RNA Sequencing Analysis

Glioma single‐cell sequencing data were obtained from the TISCH2 website [15], including datasets GSE102130, GSE131928, GSE135437, GSE148842, GSE162631, GSE163108, GSE70630, GSE84465, and GSE89567. AGTRAP and AT1R expression across different cell lines were visualized using the TISCH2 website.

### 2.3 Prognostic Analysis

Kaplan–Meier survival and Cox regression analyses were conducted using the “survival” package. A nomogram was constructed using the “rms” package, with calibration plots used to assess its predictive accuracy. The “pROC” package was used to generate receiver operating characteristic (ROC) curves to evaluate the model's prognostic performance.

### 2.4 Pathway Enrichment Analysis

As described before [16], gene set variation analysis (GSVA) was performed to explore biological functions associated with AGTRAP using the “h.all.v7.4. symbols” and “c2.cp.kegg.v7.4. symbols” datasets from the MsigDB database. Differentially expressed genes (DEGs) were analyzed via Gene Set Enrichment Analysis (GSEA) in groups with high and low AGTRAP expression. Pathways with a false discovery rate (FDR) less than 0.25 and an adjusted *p*‐value less than 0.05 were considered significantly enriched.

### 2.5 Evaluation of Tumor Immune Microenvironment

Tumor purity in gliomas was assayed using the ESTIMATE algorithm [17]. Immune cell infiltration levels were quantified using multiple computational methods, including XCELL [18], QUANTISEQ [19], CIBERSORT [20], EPIC [21], MCPCOUNTER [22], and TIMER [23]. In addition, the ssGSEA algorithm [24] was used to compute enrichment scores of tumor microenvironment‐related signatures obtained from the “IOBR” package [25].

### 2.6 Prediction of Immunotherapy Response and Chemotherapeutic Sensitivity

Expression data and clinical information from immunotherapy cohorts (GSE91061, Braun, IMvigor210, and PRJNA482620) were retrieved from the TIGER platform. Samples within each cohort were stratified by AGTRAP expression to evaluate associations with outcome/response. In addition, data from GDSC and PRISM databases were used to analyze correlations between AGTRAP expression and drug sensitivity in glioma cohorts.

### 2.7 Cell Culture

Glioma cell lines (A172 and U251) and THP‐1 cell line were provided by Renmin Hospital of Wuhan University. A172 and U251 cells were cultured in DMEM (Pricella, PM150210) supplemented with 10% fetal bovine serum (FBS, Gibco, A5256701), and 1% penicillin–streptomycin (PS, Pricella, PB180120). THP‐1 cells were cultured in PRIM1640 (Pricella, PM150110) supplemented with 10% FBS and 1% PS. THP‐1 cells were induced to differentiate into macrophages using 100 ng/mL of Phorbol 12‐myristate 13‐acetate (PMA, MCE, HY‐18739) for subsequent experiments. Cultures were maintained at 37°C in a humidified incubator containing 5% CO_2_. Besides, A172, U251, and THP‐1 cell lines underwent short tandem repeat profiling for authentication and tested negative for Mycoplasma.

### 2.8 Cell Transfection

Lentiviral vector plasmids (pLKO.1, pMD2G, and psPAX2) were obtained from Huzhou Central Hospital. The target sequences were inserted into pLKO.1 and co‐transfected into 293T cells along with pMD2G and psPAX2. The supernatant containing the virus was collected and used to infect A172 and U251 cells. Puromycin (MCE, HY‐B1743) selection was applied to generate stable AGTRAP‐knockdown cell lines. The target sequences were CGACGCCATAAGCATGTTTCT (sh1‐AGTRAP) and CGTAGTGCCTACCAGACGATT (sh2‐AGTRAP).

### 2.9 CCK8 Assay

A172 and U251 cells were seeded into 96‐well plates at a density of 1 × 10^3^ cells per well. At 0, 24, 48, and 72 h, 10% CCK8 reagent (Biosharp, BS350B) was added, and optical density (OD) values were measured at 450 nm after 30 min of incubation.

### 2.10 EdU Staining

EdU reagent (Beyotime, C0075) was added to A172 and U251 cells according to the experimental protocol, followed by a two‐hour incubation. Cells were then stained, and images were captured using a microscope. Data analysis was performed using ImageJ software.

### 2.11 Colony Formation Assay

Glioma cells (A172 and U251, 1 × 10^3^ *cells per well*) were seeded into six‐well plates. After 14 days of incubation, cells were fixed with 4% paraformaldehyde and stained with crystal violet. Colonies were visualized under a microscope and analyzed using ImageJ software.

### 2.12 Quantitative Real‐Time PCR (qRT–PCR)

Total RNA was extracted from glioma cells using TRIzol reagent (Vazyme, R411) according to the manufacturer's instructions. RNA concentration and purity were determined spectrophotometrically. Complementary DNA (cDNA) was synthesized using a reverse transcription kit with equal amounts of total RNA as input. Quantitative real‐time PCR was performed on a real‐time PCR system using a SYBR Green–based qPCR premix (Vazyme, Q713). IL‐6 expression was quantified with gene‐specific primers (Forward: ACTCACCTCTTCAGAACGAATTG; Reverse: CCATCTTTGGAAGGTTCAGGTTG) and normalized to GAPDH (Forward: TGTGGGCATCAATGGATTTGG; Reverse: ACACCATGTATTCCGGGTCAAT) as the internal reference. Each experiment was performed with at least three independent biological replicates.

### 2.13 Western Blot Analysis

A172 and U251 cells were lysed using RIPA buffer (Biosharp, BL504) containing a protease inhibitor cocktail (Biosharp, BL629). Protein concentration was determined using a BCA kit (Beyotime, P0012S). Equal amounts of protein were subjected to electrophoresis, transferred to PVDF membrane, and blocked. Membranes were incubated overnight at 4°C with primary antibodies, including anti‐AGTRAP (GeneTex, GTX64875, 1:1000), anti‐CDK2 (Beyotime, AF1063, 1:500), anti‐Bcl2 (abcam, ab182858, 1:2000), anti‐Bax (abcam, ab32503, 1:1000), anti‐cleaved‐caspase‐3 (abcam, ab2302, 1:1000), anti‐IL‐6 (abcam, ab214429, 1:1000), anti‐p‐JAK2 (CST, 8082, 1:1000), anti‐JAK2 (CST, 3230, 1:1000), anti‐p‐STAT3 (CST, 9145, 1:1000), anti‐STAT3 (CST, 9139, 1:1000), and anti‐β‐actin (abcam, ab8226, 1:2000). The following day, membranes were incubated with secondary antibodies and developed using an ECL detection system (Biorad). Image J software was used for statistical analysis.

### 2.14 Orthotopic Xenograft Tumor Model

Four‐week‐old female NOD.Cg‐Prkdcscid Il2rgtm1Wjl/SzJ (NCG) mice were obtained from the Shanghai SLAC Laboratory Animal Co. Ltd. Mice were anesthetized with isoflurane (induction 3%–4%; maintenance 1%–2% in oxygen) during stereotactic implantation. Body temperature was maintained using a heating pad. Following anesthesia, animals were secured in a stereotaxic frame. After standard aseptic preparation of the surgical site, a midline scalp incision was made to expose the skull. A burr hole was created using a high‐speed micro‐drill (Fine Science Tools, USA) at the following coordinates relative to bregma: AP −1.0 mm, ML +2.0 mm, DV −2.5 mm. U251‐luc cells in logarithmic growth phase were harvested and resuspended in serum‐free DMEM at a density of 2 × 10^5^ cells/μL. Using a 5 μL Hamilton syringe, 2 μL of cell suspension was slowly infused into striatum. The surgical wound was closed. Tumor growth was monitored weekly for 28 days using in vivo bioluminescence imaging (IVIS Spectrum, PerkinElmer). Following the experimental endpoint, mice were euthanized by cervical dislocation under deep anesthesia, and brain tissues were harvested for subsequent hematoxylin and eosin (H&E) histological analysis.

### 2.15 Tunel Staining

The paraffin‐embedded tissue sections were first baked, followed by dewaxing in xylene and rehydration through a graded alcohol series. Sections were then permeabilized with proteinase K. The TUNEL (Elabscience, E‐CK‐A320) reaction mixture was prepared according to the manufacturer's protocol and applied to the sections, which were then incubated at 37°C in a dark humidified chamber for 60 min. After incubation, the sections were washed three times with PBS buffer to stop the reaction. Nuclei were counterstained with DAPI, and the sections were mounted using an anti‐fade mounting medium. Fluorescence images were captured using a fluorescence microscope, and quantitative analysis was performed using Image J software.

### 2.16 Immunofluorescence

Tissue sections underwent standard processing including heat immobilization at 60°C, xylene‐mediated dewaxing, and rehydration through a graded ethanol series (100%–70%). Antigen retrieval was subsequently performed using citrate buffer (pH 6.0) followed by membrane permeabilization with 0.1% Triton X‐100 and blocking in 5% BSA. Primary antibody incubation was conducted at 4°C under light‐protected conditions overnight, including anti‐Ki67 (abcam, ab16667, 1:200) and anti‐IBA1 (1:100). Following three PBS washes (5 min each), sections were incubated with Alexa Fluor‐conjugated secondary antibodies (1:500) for 2 h at ambient temperature protected from light. Nuclear counterstaining was achieved using DAPI‐containing mounting medium. Fluorescence images were captured using a fluorescence microscope, and quantitative analysis was performed using ImageJ software.

### 2.17 Immunohistochemistry and Immunostaining Score (ISS)

Following paraffin embedding, glioma tissue samples were sectioned at 5 μm thickness. The sections were dried at 65°C for 1 h, dewaxed in xylene, and rehydrated through a graded ethanol series. Antigen retrieval was conducted via microwave treatment. Subsequently, endogenous peroxidase activity was quenched with 3% H₂O₂, and nonspecific binding was blocked with 5% BSA for 30 min at room temperature. Primary antibody incubation was carried out overnight at 4°C, including anti‐AGTRAP (1:200) and anti‐IBA1 (CST, 17198, 1:300). After PBS washing, HRP‐conjugated secondary antibody was applied, and immunoreactivity was detected using DAB substrate. Then, sections were counterstained with hematoxylin, differentiated in acid alcohol, dehydrated, cleared, and mounted with neutral balsam. Finally, the protein expression levels of AGTRAP and IBA1 were quantitatively evaluated using the ISS, calculated as follows: ISS = PS × IS, where: PS (Percentage Score) represents the proportion of immunopositive cells, graded as: 0 (< 10%), 1 (10%–25%), 2 (26%–50%), 3 (51%–75%), or 4 (> 75%). IS (Intensity Score) reflects staining intensity, categorized as: 0 (negative), 1 (weak), 2 (moderate), or 3 (strong).

### 2.18 Transwell Assay

A co‐culture system was established using Transwell chambers. PMA‐induced THP‐1 cells were seeded in the upper chamber, while glioma cells (A172 or U251) were placed in the lower chamber. After 24 h of co‐culture, cells were fixed and stained with crystal violet. Migrated cells on the lower surface of the membrane were counted under a microscope to assess the chemotactic effect.

### 2.19 Flow Cytometry

A172 and U251 cells were collected, resuspended in PBS, and centrifuged. Cells were stained with a working solution containing FITC‐Annexin V and PE‐PI, incubated in the dark for 30 min, and analyzed by flow cytometry (BD FACSCantoll).

### 2.20 Statistical Analysis

All statistical analyses were performed using R software (version 4.0.2). Normally distributed variables were analyzed using *t*‐tests and one‐way ANOVA. Categorical variables were compared using Fisher's exact test or chi‐square test. Correlations were assessed using Spearman's or Pearson's method. Survival differences were evaluated using Kaplan–Meier analysis and log‐rank tests. Hazard ratios and confidence intervals were calculated using univariate and multivariate Cox regression. Statistical significance was set at *p* < 0.05.

## 3 Results

### 3.1 AGTRAP Expression Characteristics in Gliomas

**FIGURE 1:**
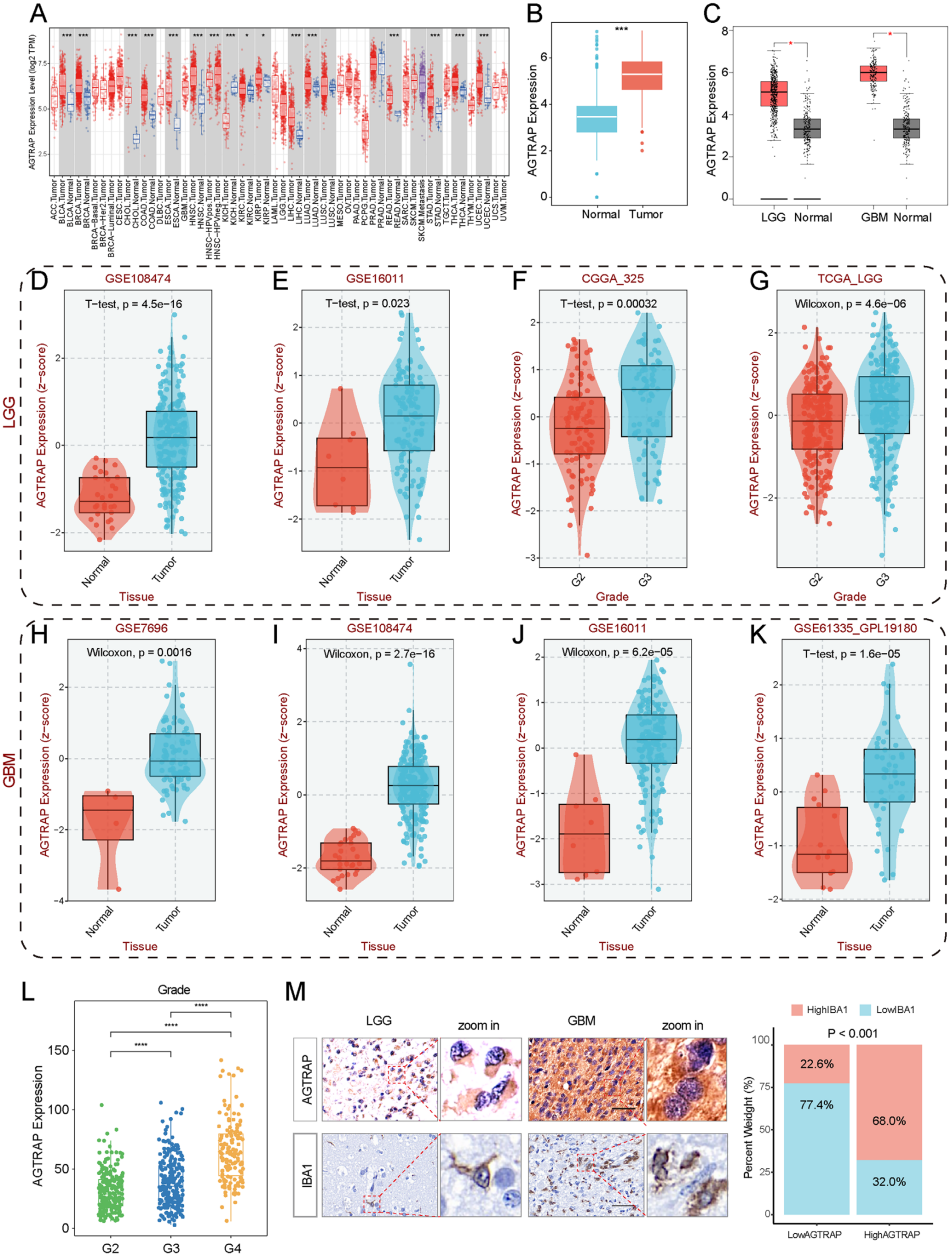
The expression characteristics of AGTRAP in gliomas. (A) The expression of AGTRAP in pan‐cancer. (B) The different expression of AGTRAP between normal and tumor tissues in TCGA cohort. (C) The boxplot showed that AGTRAP had a higher level in both LGG and GBM. (D, E) The different expression level of AGTRAP in LGG patients in GSE108474 and GSE16011 cohorts. (F, G) The AGTRAP had a higher level in G3 than in G2. (H–K) The different expression level of AGTRAP in GBM patients in GSE7696, GSE108474, GSE16011, and GSE61335. (L) The AGTRAP had a higher level in higher grade in TCGA cohort. (M) The representative images of AGTRAP and IBA1 among different grades in immunohistochemistry, scale bar, 50 μm.

To investigate AGTRAP expression across various tumor types, we analyzed its level in 32 tumor categories using data from The Cancer Genome Atlas (TCGA). Our findings indicated that AGTRAP expression was elevated in most tumor tissues compared to normal tissues, except for prostate adenocarcinoma (PRAD) and kidney chromophobe (KICH) (Figure 1A). In addition, we extracted data from normal brain and glioma tissues from the Genotype‐Tissue Expression (GTEx) and TCGA datasets. The analysis revealed significantly higher AGTRAP expression in glioma tissues than in normal brain tissues (Figure 1B). Recognizing the heterogeneity between LGG and GBM, we stratified the entire glioma dataset into two separate cohorts for parallel analysis, which yielded consistent results for both LGG and GBM (Figure 1C).

Further validation was conducted using LGG and GBM datasets from GEO databases, where upregulated AGTRAP expression was observed in datasets GSE108474, GSE16011, GSE7696, and GSE61335, confirming stable alterations in expression levels in both LGG (Figure 1D,E) and GBM (Figure 1H–K). Analysis of the CGGA325 and TCGA cohorts showed that AGTRAP expression increases with tumor grade (Figure 1F,G,L). Immunohistochemistry confirmed this trend (Figure 1M), suggesting a potential link between AGTRAP expression and tumor progression.

We further analyzed the correlation between AGTRAP expression and established clinical and molecular characteristics in both the LGG and GBM cohorts. Higher AGTRAP expression was observed in the older glioma patients (Figure S1F). In CGGA301, CGGA325, CGGA693, and E‐MTAB‐3892 datasets, patients with a 1p/19q co‐deletion exhibited significantly lower AGTRAP expression in both GBM (Figure S1A,B) and LGG (Figure S1G–J). In addition, AGTRAP expression correlated with isocitrate dehydrogenase (IDH) mutations in both GBM (Figure S1C–E) and LGG (Figure S1K,L). Notably, AGTRAP expression varied among histopathological subtypes of LGG (Figure S1M). These findings suggest that AGTRAP may serve as a critical clinical prognostic marker for glioma progression.

### 3.2 Prognostic Value and Predictive Ability of AGTRAP

**FIGURE 2:**
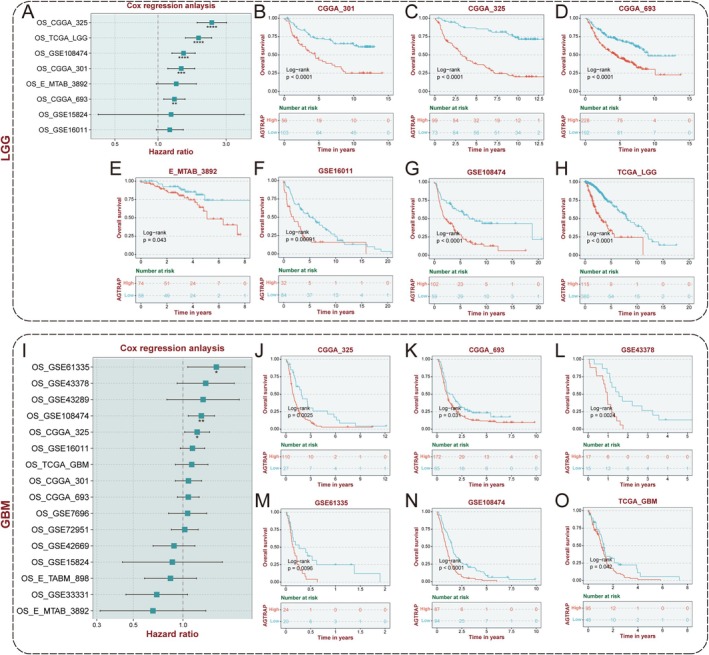
The prognostic value of AGTRAP. (A) The forest plot showed univariate Cox analyses of AGTRAP in LGG patients in eight independent cohorts. (B–H) There were significantly different survival rates between high and low AGTRAP patients with LGG. (I) The forest plot showed univariate Cox analyses of AGTRAP in GBM patients in eight independent cohorts. (J–O) There were significantly different survival rates between high and low AGTRAP patients with GBM.

Patients diagnosed with LGG exhibited a more favorable prognosis compared to those with GBM [4]. To assess the prognostic significance of AGTRAP, we conducted univariate Cox regression and survival curve analyses in LGG and GBM cohorts. Univariate Cox analysis identified AGTRAP as a significant risk factor across multiple datasets, including CGGA325, CGGA301, TCGA, GSE108474, and CGGA693 (Figure 2A). Survival curve analyses conducted on CGGA301, CGGA325, CGGA693, E‐MTAB‐3892, GSE16011, GSE108474, and TCGA cohorts consistently confirmed the prognostic value of AGTRAP (Figure 2B–H). Similarly, parallel analyses of multiple independent GBM cohorts yielded consistent results (Figure 2I–O), indicating that elevated AGTRAP expression is strongly associated with poor outcomes in gliomas.

Furthermore, receiver operating characteristic (ROC) curve analysis applied to TCGA glioma cohort demonstrated that AGTRAP has a robust prognostic predictive capability (Figure S2A). Both univariate and multivariate Cox analyses confirmed that AGTRAP was an independent prognostic factor for gliomas (Figure S2B,C). In addition, we developed a nomogram integrating AGTRAP mRNA expression with established clinical parameters (Figure S2D). Calibration and ROC curves validated the accuracy and superior performance of the nomogram (Figure S2E,F). Decision curve analysis further demonstrated that the nomogram model provided the highest net benefit (Figure S2G–I).

### 3.3 Role of AGTRAP in Apoptosis and Immune Pathways

**FIGURE 3:**
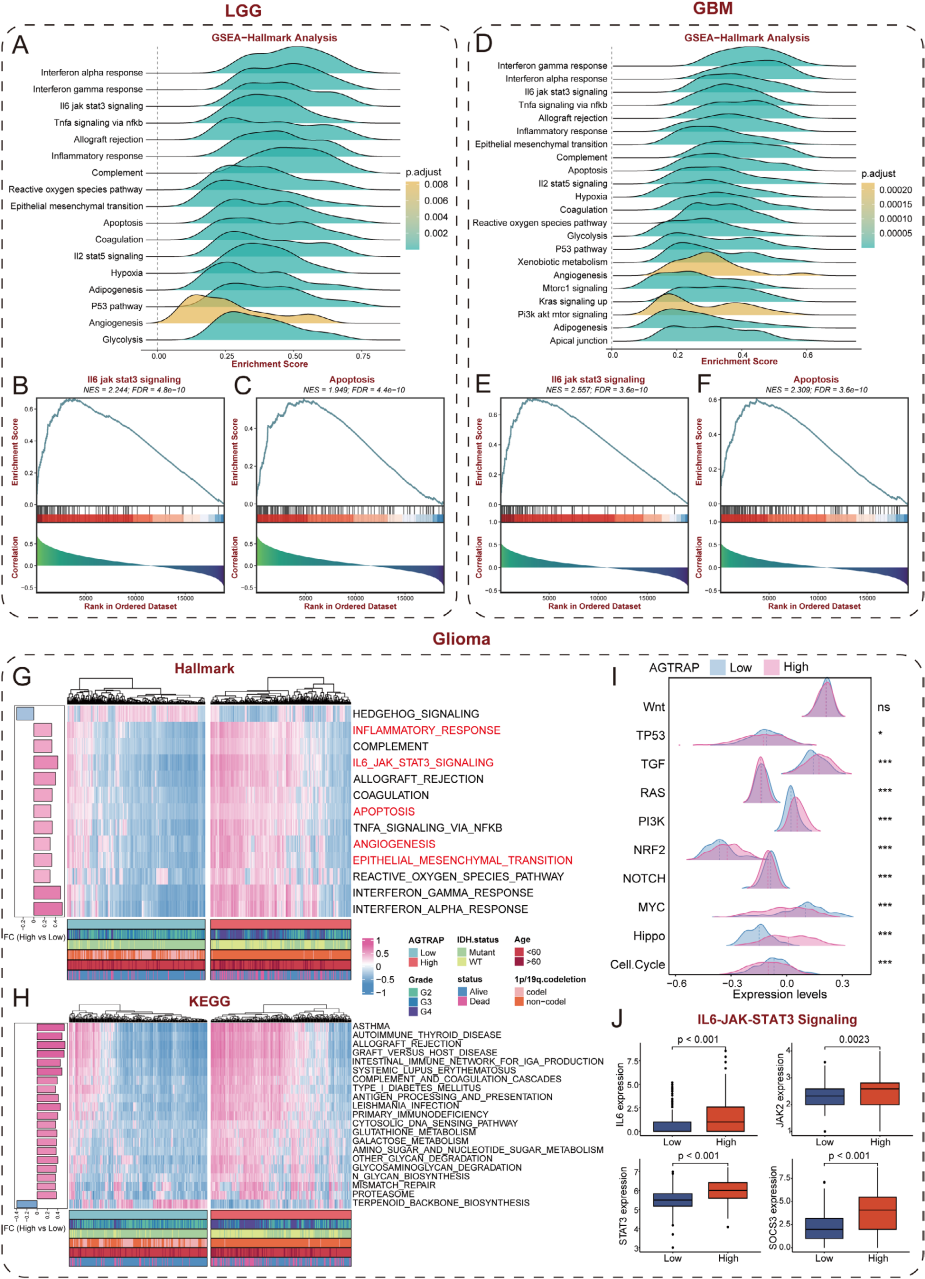
Biological function analyses associated with AGTRAP in glioma. (A) Overview of significantly enriched Hallmark pathways in LGG stratified by AGTRAP expression. (B, C) GSEA plots showing enrichment of the IL‐6/JAK/STAT3 signaling pathway (B) and the apoptosis pathway (C) in the AGTRAP‐high group in the LGG cohort. (D) Overview of significantly enriched Hallmark pathways in GBM stratified by AGTRAP expression. (E, F) GSEA plots showing enrichment of the IL‐6/JAK/STAT3 signaling pathway (E) and the apoptosis pathway (F) in the AGTRAP‐high group in the GBM cohort. (G, H) GSVA results comparing pathway activity between AGTRAP‐high and AGTRAP‐low groups in the TCGA‐glioma cohort using Hallmark (G) and KEGG (H) gene sets. (I) Comparison of activity scores for 10 widely recognized cancer‐related signaling pathways between AGTRAP‐high and AGTRAP‐low groups. (J) Expression levels of key genes in the IL‐6/JAK2/STAT3 signaling axis (including IL‐6, JAK2, STAT3, and SOCS3) between AGTRAP‐high and AGTRAP‐low groups.

To explore biological programs associated with AGTRAP in glioma, we stratified LGG and GBM samples into AGTRAP‐high and AGTRAP‐low groups (median cutoff) and performed differential expression followed by GSEA. Across both LGG and GBM cohorts, Hallmark analysis consistently highlighted enrichment of IL‐6/JAK/STAT3 signaling together with apoptosis‐ and immune‐related pathways in AGTRAP‐high tumors (Figure 3A–F). These associations were reproduced in the combined TCGA glioma cohort using GSVA across Hallmark and KEGG collections, supporting a coherent link between AGTRAP expression and inflammatory/survival programs (Figure 3G,H).

At single‐cell resolution, AGTRAP transcripts were enriched in malignant cells as well as monocyte/macrophage populations, suggesting a role in glioma progression through interactions with immune and tumor cell populations (Figure S3A,C). However, AGTR1 (AT1R) transcripts were scarcely detected across the glioma ecosystem and were essentially absent in malignant cells (Figure S3B,C). Additionally, comparative oncogenic pathway analysis between high‐ and low‐AGTRAP expression groups demonstrated significant enrichment of TGF, PI3K, NRF2, and Hippo signaling pathways in the high‐AGTRAP group (Figure 3I).

Given the recurrent enrichment of IL‐6/JAK/STAT3 signatures, we further examined core components of this pathway. IL‐6, JAK2, STAT3, and SOCS3 displayed higher expression in AGTRAP‐high tumors, supporting IL‐6–linked JAK/STAT3 signaling as a candidate downstream program associated with AGTRAP (Figure 3J).

### 3.4 Effect of AGTRAP Knockdown on Glioma Cells

**FIGURE 4:**
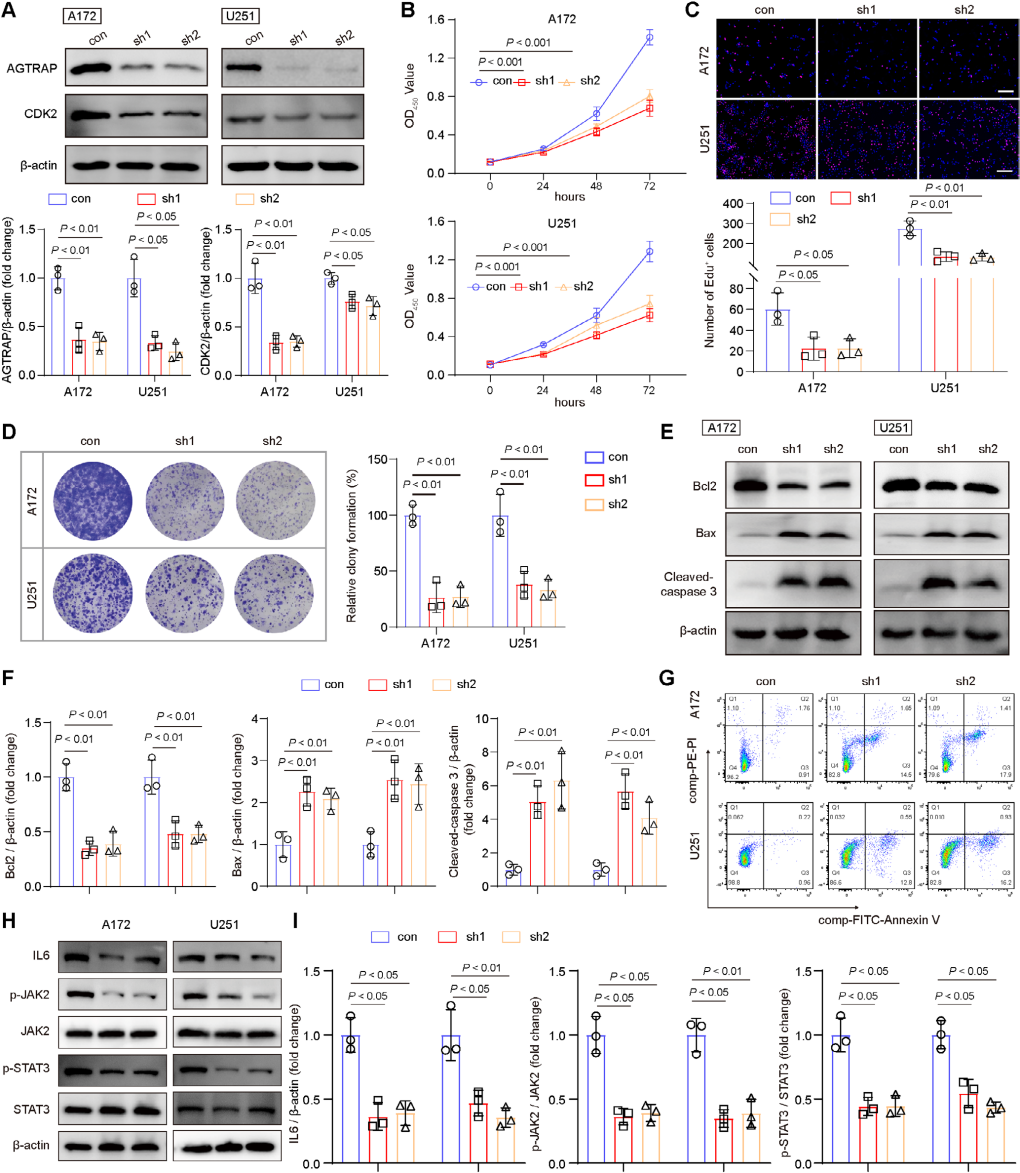
The effect of AGTRAP knockdown on glioma cells. (A) Western blot analysis of AGTRAP and CDK2 in A172 and U251 cells, *n* = 3. (B) CCK8 assay of A172 and U251 cells at 0, 24, 48, and 72 h, *n* = 3. (C) EdU staining of A172 and U251 cells, *n* = 3, scale bar, 50 μm. (D) Clone formation experiment of A172 and U251 cells, *n* = 3. (E, F) Western blot analysis of Bcl2, Bax, and cleaved‐caspase3 in A172 and U251 cells, *n* = 3. (G) Flow Cytometry analysis of A172 and U251 cells, *n* = 3. (H, I) Western blot analysis of IL‐6, p‐JAK2, JAK2, p‐STAT3, and STAT3 in A172 and U251 cells, *n* = 3. All values represent SD ± mean.

To comprehensively investigate the role of AGTRAP on glioma cells, we engineered sh1‐AGTRAP (sh1) and sh2‐AGTRAP (sh2) constructs to suppress AGTRAP expression in A172 and U251 cell lines. Western blot analysis confirmed that both sh1 and sh2 significantly reduced the levels, along with the proliferation‐associated protein CDK2 (Figure 4A).

To assess the effects of AGTRAP on glioma cell proliferation, we conducted a CCK8 assay. Compared to the control (con) group, AGTRAP knockdown resulted in a significant decrease in OD values in A172 and U251 cells at 72 h (Figure 4B). Similarly, EdU staining revealed a reduction in EdU‐positive cells in the sh1 and sh2 groups in both cell lines (Figure 4C). Furthermore, colony formation assays demonstrated that AGTRAP knockdown led to a decrease in glioma cell colony formation (Figure 4D).

We also examined the effect of AGTRAP knockdown on apoptosis in A172 and U251 cells. Western blot analysis indicated that AGTRAP knockdown reduced Bcl2 levels while increasing Bax and cleaved‐caspase 3 levels, suggesting an induction of apoptosis (Figure 4E,F). Flow cytometry analysis further supported these findings, showing a higher percentage of apoptotic cells in the sh1 and sh2 groups compared to the control group (Figure 4G and Figure S4A).

**FIGURE 5:**
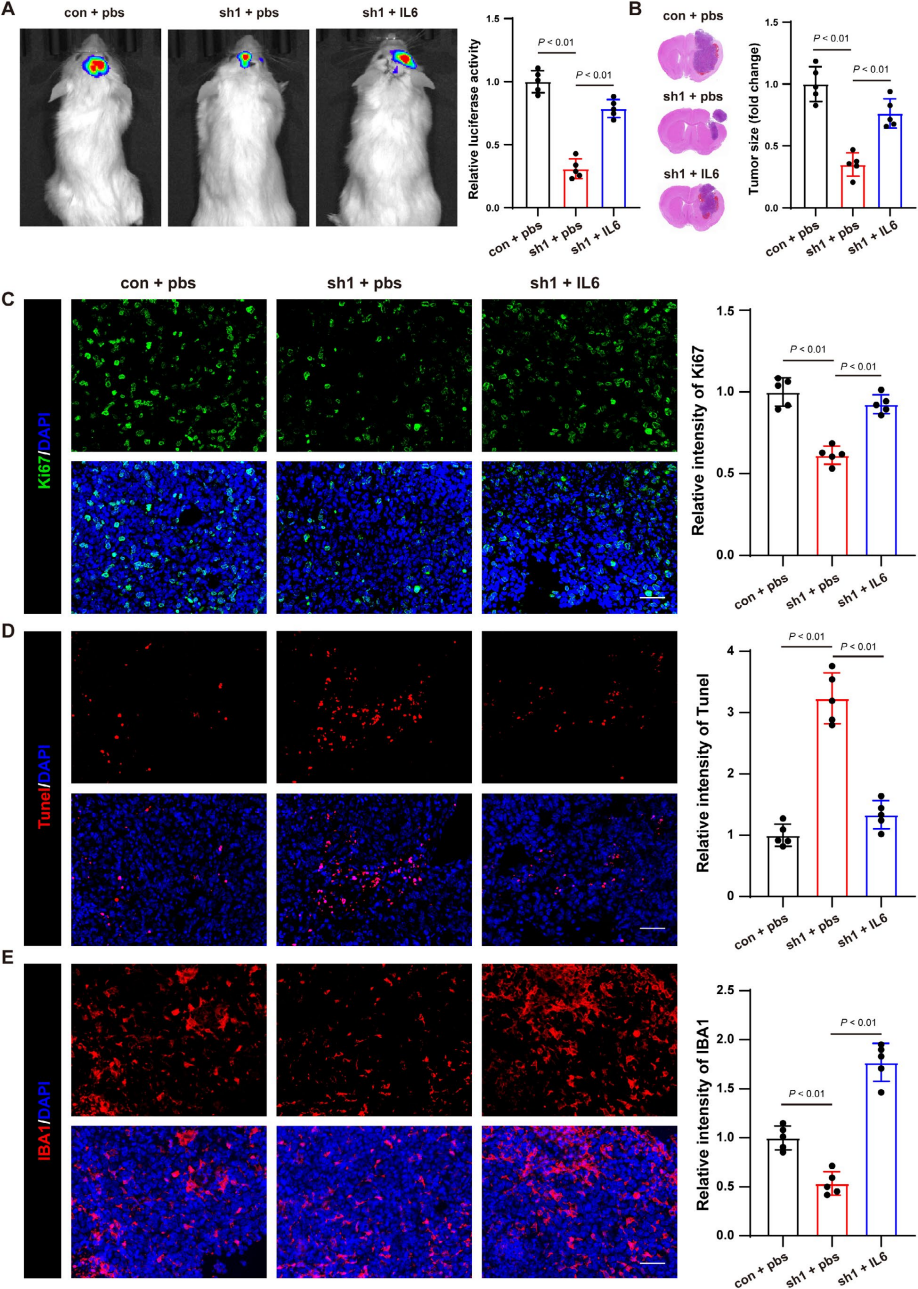
The Effects of AGTRAP on glioma growth in vivo. (A) In vivo bioluminescence imaging (BLI) was used to monitor intracranial tumor growth in NCG mice. (B) Quantification of BLI signal. (C) Tumor volume and (D) tumor weight. (E) Representative immunofluorescence staining of AGTRAP, Ki67, and IBA1 in orthotopic tumors. (F) Quantification of Ki67‐positive cells and IBA1 signal. Scale bar = 50 μm. All values represent mean ± SD.

To assess the role of AGTRAP in tumorigenicity, we established an orthotopic xenograft tumor model using U251‐luc cells. The result demonstrated a reduction in bioluminescence signal intensity in the AGTRAP‐knockdown group compared to the control group, accompanied by a decrease in tumor tissue volume (Figure 5A,B). Immunostaining further revealed a lower Ki67 fluorescence intensity in AGTRAP‐knockdown tumor cells, indicating reduced proliferation, whereas TUNEL staining showed an increase in apoptosis (Figure 5C,D). These findings suggest that AGTRAP knockdown suppresses glioma progression by modulating cell proliferation and apoptosis in vivo.

To further investigate the connection between AGTRAP and the IL‐6/JAK2/STAT3 signaling pathway, we assessed the expression levels of IL‐6, p‐JAK2, JAK2, p‐STAT3, and STAT3. Western blot analysis showed that AGTRAP knockdown led to reduced IL‐6 expression and phosphorylation of JAK2 and STAT3 (Figure 4H,I).

To clarify the regulatory level of IL‐6, we quantified IL‐6 transcripts by qRT–PCR and IL‐6 secretion by ELISA, and observed that IL‐6 transcription and secretion were significantly reduced after AGTRAP knockdown (Figure S5A,B), consistent with the reduction in IL‐6 protein. However, the phosphorylation status of NF‐κB p65 was not detectably altered by AGTRAP silencing under our experimental conditions (Figure S6A,B), suggesting that IL‐6 downregulation in this setting is not primarily driven by canonical NF‐κB activation.

### 3.5 IL‐6/JAK2/STAT3 Signaling Mediates AGTRAP'S Effects on Glioma Cell Proliferation and Apoptosis

**FIGURE 6:**
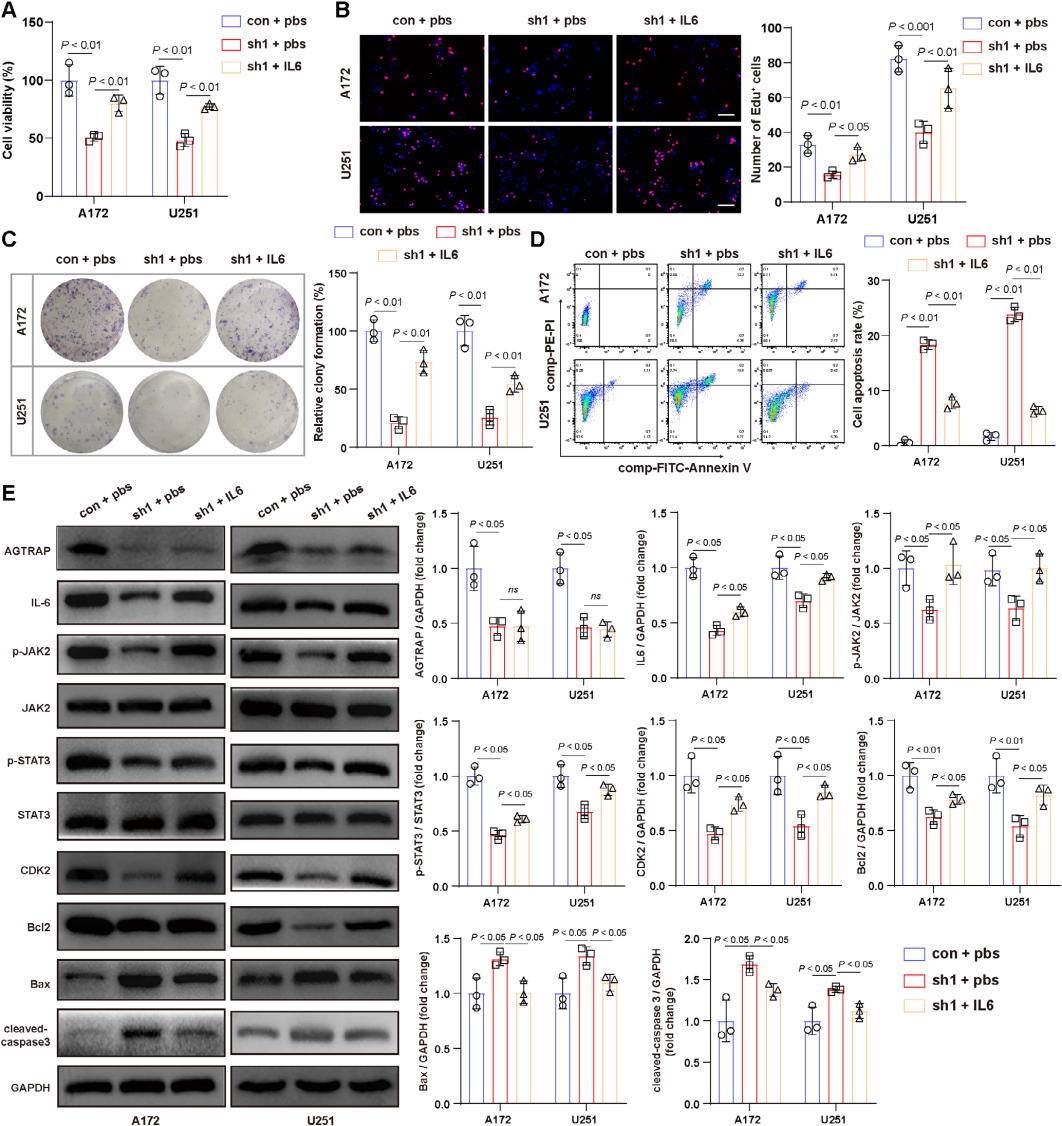
IL‐6/JAK2/STAT3 signaling mediates the effects of AGTRAP on the proliferation and apoptosis of glioma cells. (A) CCK‐8 assay of A172 and U251 cells. (B) EdU staining and quantification of proliferating cells. (C) Colony formation assay. (D) Flow cytometry analysis of apoptosis. (E) Representative western blotting of IL‐6/JAK2/STAT3 signaling components after AGTRAP knockdown and after recombinant IL‐6 treatment. (F) Quantification of p‐JAK2/JAK2 and p‐STAT3/STAT3 ratios. (G) Apoptosis‐related proteins by western blotting. *n* = 3 biological replicates. All values represent mean ± SD.

To determine whether the IL‐6/JAK2/STAT3 signaling pathway mediates the effects of AGTRAP on glioma cells, we treated A172 and U251 with recombinant IL‐6. The CCK8 assay demonstrated that IL‐6 treatment significantly enhanced the viability of A172 and U251 cells compared to that in the AGTRAP‐knockdown group (Figure 6A). Similarly, EdU staining showed an increase in EdU‐positive cells after IL‐6 treatment (Figure 6B), and colony formation assays revealed an increase in the number of colonies formed by A172 and U251 cells in the IL‐6 treatment group (Figure 6C).

Flow cytometry analysis indicated that IL‐6 treatment reduced the percentage of apoptotic cells in AGTRAP‐knockdown glioma cells (Figure 6D). Western blot analysis further demonstrated that IL‐6 treatment regulated IL‐6, p‐JAK2, p‐STAT3, CDK2, and Bcl‐2 while downregulating Bax and cleaved‐caspase‐3 levels in A172 and U251 cells (Figure 6E). However, IL‐6 treatment did not alter AGTRAP expression (Figure 6E).

To validate these findings in vivo, we administered IL‐6 to mice transplanted with AGTRAP‐knockdown glioma cells. IL‐6 treatment resulted in an increase in dual‐luciferase activity and tumor volume (Figure 5A,B). Additionally, immunofluorescence staining showed that IL‐6 treatment elevated Ki67 intensity in glioma cells, indicating increased proliferation, while TUNEL staining demonstrated a reduction in the number of apoptotic cells (Figure 5C,D).

### 3.6 AGTRAP Alters the Tumor Immune Microenvironment and Glioma Genomic Landscape

**FIGURE 7:**
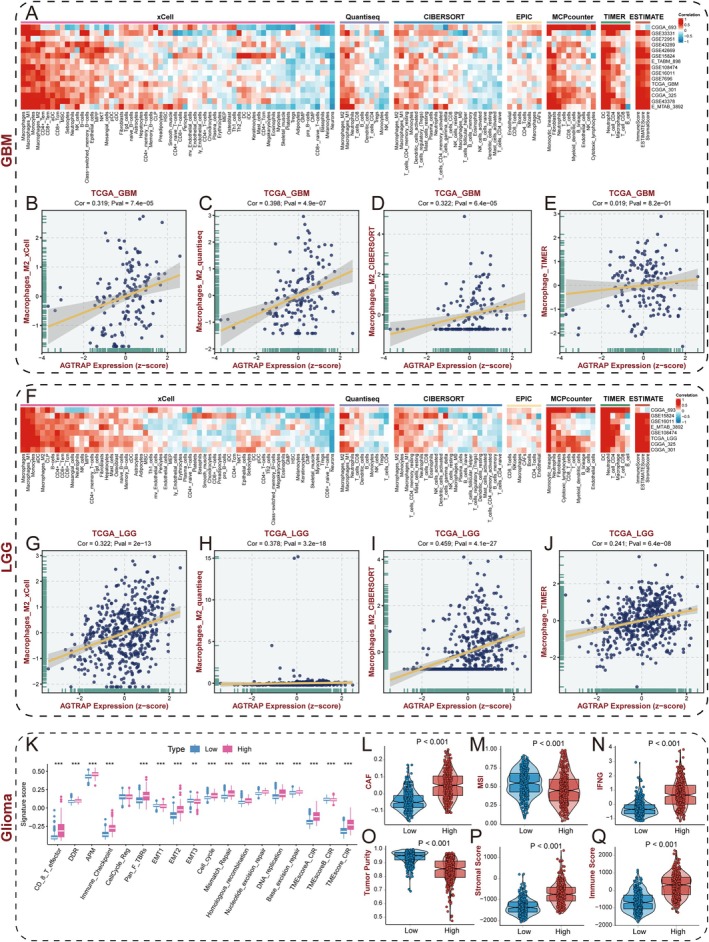
The immune characteristics of AGTRAP in gliomas. (A) Heatmap showing correlations between AGTRAP expression and immune cell infiltration estimates across 15 GBM cohorts. (B–E) Scatterplots showing correlations between AGTRAP expression and inferred M2 macrophage infiltration in TCGA‐GBM using xCell (B), quanTIseq (C), CIBERSORT (D), and TIMER (E). (F) Heatmap showing correlations between AGTRAP expression and immune cell infiltration estimates across eight LGG cohorts. (G–J) Scatterplots showing correlations between AGTRAP expression and inferred M2 macrophage infiltration in TCGA‐LGG using xCell (G), quanTIseq (H), CIBERSORT (I), and TIMER (J). (K) Comparison of TME‐related signature scores between AGTRAP‐high and AGTRAP‐low groups. (L–Q) Differences in CAF (L), MSI (M), IFNG (N), tumor purity (O), stromal score (P), and immune score (Q) between AGTRAP‐high and AGTRAP‐low groups.

Based on the enrichment of immune‐related programs in AGTRAP‐high tumors, we next examined the relationship between AGTRAP expression and immune/stromal features in glioma. To increase robustness, we applied multiple deconvolution approaches (TIMER, CIBERSORT, ESTIMATE, quanTIseq, MCP‐counter, xCell, and EPIC) across GBM and LGG cohorts (Figure 7A,F).

Across both LGG and GBM, AGTRAP expression was positively correlated with ESTIMATE immune and stromal scores, indicating that AGTRAP‐high tumors tend to harbor increased non‐tumor components. Immune deconvolution further showed that macrophage‐related signatures were the most consistently associated with AGTRAP expression across multiple algorithms and independent cohorts (Figure 7B–E,G–J), supporting a myeloid‐enriched microenvironment in AGTRAP‐high gliomas. To corroborate these in silico observations at the tissue level, we assessed macrophage/microglia markers in tumor sections. In glioma tissue microarrays, AGTRAP expression was positively associated with the number of IBA1‐positive cells (Figure 1M). Consistently, in the orthotopic xenograft model (NCG mice), AGTRAP knockdown was accompanied by reduced IBA1 fluorescence intensity in tumor tissue, whereas recombinant IL‐6 increased the IBA1 signal (Figure 5E). We further performed an in vitro chemotaxis assay using a Transwell co‐culture system to evaluate glioma cell‐driven recruitment of macrophage. AGTRAP knockdown in glioma cells resulted in a decreased migration rate of PMA‐induced THP‐1 cells compared with controls (Figure S7A,B).

In the TCGA glioma cohort, AGTRAP‐high tumors also displayed higher scores for several tumor microenvironment (TME)‐related signatures and stromal features (Figure 7K), including cancer‐associated fibroblast (CAF) signatures and interferon‐γ (IFNG)‐related signals (Figure 7L–Q). These metrics reflect the complex inflammatory and stromal composition of glioma and should be interpreted as correlates rather than direct evidence of immune activation or therapeutic response.

We further explored whether AGTRAP expression tracked with genomic features in TCGA glioma. The AGTRAP‐high group exhibited a higher mutational burden than the AGTRAP‐low group (Figure S8A–C). IDH1, CIC, and FUBP1 alterations were enriched in the AGTRAP‐low group, whereas PTEN, EGFR, TTN, and TP53 alterations were more frequent in the AGTRAP‐high group (Figure S8D). In addition, regardless of the glioma grade, AGTRAP expression positively correlated with immune escape‐associated genes such as PTEN and EGFR (Figure S8E).

### 3.7 AGTRAP Predicts Chemotherapeutic Sensitivity and Shows Exploratory Associations With Immunotherapy Outcomes

**FIGURE 8:**
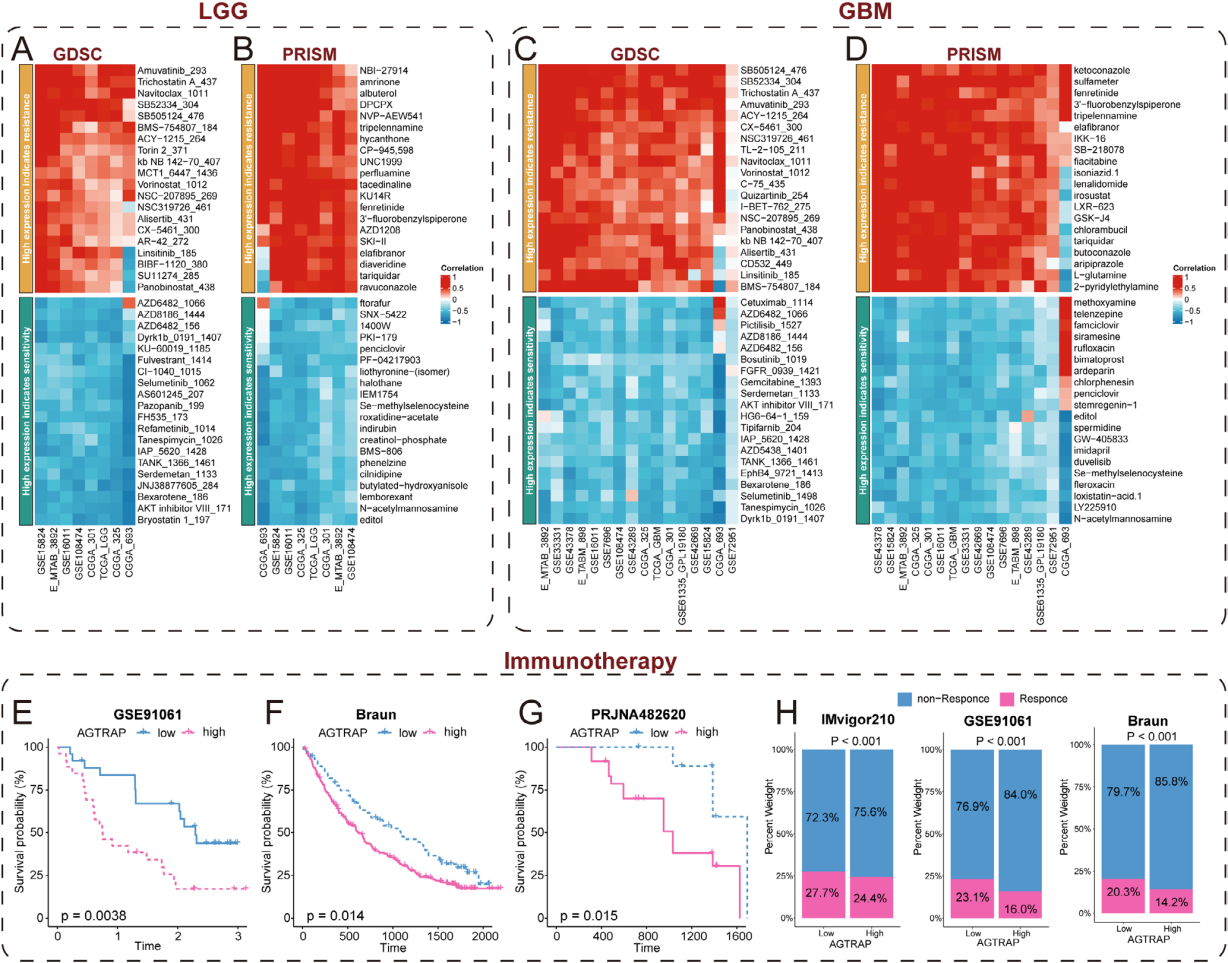
The drug sensitivity and immunotherapy response prediction ability of AGTRAP. (A, B) Heatmaps showing correlations between predicted IC50 values of chemotherapeutic agents and AGTRAP expression in LGG using the GDSC (A) and PRISM (B) resources. (C, D) Heatmaps showing correlations between predicted IC50 values and AGTRAP expression in GBM using GDSC (C) and PRISM (D). (E–G) Kaplan–Meier survival analyses stratified by AGTRAP expression in immune checkpoint blockade cohorts: GSE91061 (E; melanoma), Braun (F; renal cell carcinoma), and PRJNA482620 (G; anti–PD‐1–treated glioblastoma). (H) Stacked bar plots comparing response categories between AGTRAP‐high and AGTRAP‐low groups in IMvigor210 (urothelial carcinoma), GSE91061 (melanoma), and the Braun cohort (renal cell carcinoma). Analyses based on non‐glioma cohorts are presented as exploratory, cross‐tumor associations.

To assess the relationship between AGTRAP expression and treatment‐related phenotypes, we first analyzed its association with predicted chemotherapeutic sensitivity using GDSC and PRISM resources. In both LGG and GBM cohorts, AGTRAP expression correlated with the estimated half‐maximal inhibitory concentrations (IC50) of multiple agents (Figure 8A–D), supporting a potential link between AGTRAP‐associated transcriptional states and drug‐response profiles.

We also explored whether AGTRAP expression tracked with immune checkpoint blockade (ICB) outcomes in publicly available cohorts with matched transcriptomes. These datasets include urothelial carcinoma (IMvigor210), melanoma (GSE91061), renal cell carcinoma (Braun), and an anti–PD‐1–treated glioblastoma cohort (PRJNA482620). Across the non‐glioma cohorts, lower AGTRAP expression was associated with improved survival and/or higher response rates (Figure 8E–H). Given the distinct, myeloid‐dominant and often T‐cell–excluded immune microenvironment of glioma, these cross‐tumor analyses are presented as exploratory and do not constitute glioma‐specific immunotherapy validation.

## 4 Discussion

This study identifies AGTRAP as a clinically relevant and functionally active factor in glioma. Across multiple cohorts, AGTRAP expression was elevated in glioma compared with non‐tumor brain and tracked with clinicomolecular features, including tumor grade and key molecular alterations. Functionally, AGTRAP silencing suppressed glioma cell proliferation, increased apoptosis, and reduced tumor burden in an orthotopic xenograft model. Mechanistically, we link AGTRAP to an IL‐6–associated JAK2/STAT3 survival program: AGTRAP knockdown reduced IL‐6 at both the mRNA and protein levels, attenuated JAK2/STAT3 phosphorylation, and recombinant IL‐6 partially rescued STAT3 activation and the survival phenotype. In addition, ELISA analysis of culture supernatants showed reduced secreted IL‐6 after AGTRAP knockdown, aligning extracellular cytokine output with the transcript‐level change. Together, these results support AGTRAP as a tumor cell–intrinsic modulator of inflammatory survival signaling in glioma.

AGTRAP is commonly described in cardiovascular and renal systems as a negative regulator of Ang II–AT1R signaling, where it promotes AT1R trafficking/internalization and limits inflammation [7, 8, 26, 27]. In glioma, our data point to a different context. In our single‐cell dataset, AGTR1 (AT1R) transcripts were barely detectable across the tumor ecosystem and essentially absent in malignant glioma cells, making a tumor cell–intrinsic AT1R‐dependent explanation unlikely in this setting. We therefore interpret AGTRAP's pro‐survival phenotype in glioma as more plausibly mediated through AT1R‐independent functions. This interpretation is consistent with reports that AGTRAP can engage alternative interaction partners—for example, facilitating TfR1 internalization—supporting a broader role in membrane trafficking and stress biology beyond AT1R [8]. Regarding how AGTRAP controls IL‐6, our qRT–PCR results show that IL‐6 transcripts decline following AGTRAP knockdown, indicating regulation at the transcript level (transcription and/or mRNA turnover) rather than an effect restricted to secretion. Because NF‐κB is a canonical driver of IL‐6, we assessed NF‐κB activation and observed no detectable change in p‐NF‐κB p65 or total NF‐κB p65 under our experimental conditions. These data argue against canonical NF‐κB activation as the primary route linking AGTRAP to IL‐6 in our model. One plausible explanation is that AGTRAP supports a self‐reinforcing inflammatory state in glioma cells, where IL‐6 availability and STAT3 activity participate in positive feedback that maintains cytokine expression programs [28, 29, 30]. In addition, IL‐6 mRNA is well known to be subject to AU‐rich element–dependent post‐transcriptional regulation, raising the possibility that AGTRAP‐associated trafficking or stress pathways influence IL‐6 mRNA stability through NF‐κB‐independent routes [31]. While the precise upstream link remains to be elucidated, our data support an IL‐6–linked JAK2/STAT3 survival axis downstream of AGTRAP and justify prioritizing upstream mechanistic mapping in future studies. Notably, the IL‐6 add‐back experiments provide functional support for pathway directionality by reactivating STAT3 signaling and partially reversing the survival phenotype after AGTRAP knockdown, which is consistent with an IL‐6–dependent component of AGTRAP‐driven signaling.

Our immune analyses further indicate that AGTRAP expression is associated with a myeloid/macrophage‐enriched tumor microenvironment. Across multiple deconvolution algorithms and cohorts, AGTRAP correlated most consistently with macrophage‐related signatures. This association is biologically compatible with established knowledge that gliomas are typically dominated by resident microglia and infiltrating monocyte‐derived macrophages, which often adopt immunoregulatory programs that shape disease progression and therapeutic resistance [32, 33]. Consistent with these associations, we observed that AGTRAP knockdown reduced THP‐1 migration in a Transwell co‐culture/chemotaxis setting, supporting a role for tumor‐cell AGTRAP in shaping myeloid recruitment cues. Importantly, our functional perturbations were performed in glioma cells, and therefore do not resolve whether macrophage/microglia–intrinsic AGTRAP contributes directly to polarization states. Accordingly, while tumor‐cell AGTRAP–driven IL‐6 could plausibly act in a paracrine manner to facilitate myeloid recruitment and potentially bias immunoregulatory (M2‐like) programs, macrophage/microglia‐intrinsic AGTRAP functions were not directly tested and cannot be concluded from the current experiments. Given the prominent single‐cell expression of AGTRAP in both malignant and myeloid compartments, compartment‐resolved approaches (e.g., myeloid‐targeted perturbation and spatial profiling) will be needed to determine functional dominance. In addition, because the orthotopic xenograft experiments were performed in NCG mice that lack functional T, B, and NK cells, IBA1 readouts in this setting should be interpreted as descriptive evidence of myeloid presence rather than adaptive immune regulation or checkpoint blockade resistance; therefore, immunotherapy‐related observations are best viewed as hypothesis‐generating and will require validation in immunocompetent glioma models and/or well‐annotated GBM immunotherapy cohorts.

In conclusion, our data support AGTRAP as a glioma‐relevant factor that promotes tumor cell survival through an IL‐6–linked JAK2/STAT3 program and is associated with macrophage/microglia‐related microenvironmental features. Future glioma‐centered studies using immunocompetent models and compartment‐resolved profiling will be important to define the upstream regulation of IL‐6 and to clarify the relative contributions of tumor versus myeloid AGTRAP in vivo.

## Funding

This work was supported by the public welfare application project of Huzhou (2023GZB01 to Zhongzhou Su).

## Ethics Statement

All animal procedures were approved by the Animal Care and Use Committee of Huzhou Central Hospital (Approval No. 202411003). Human glioma specimens were obtained from Renmin Hospital of Wuhan University. The study involving human participants was approved by the Institutional Ethics Committee of Renmin Hospital of Wuhan University (Approval No. WDRY2023‐K125). Written informed consent was obtained from all patients prior to sample collection.

## Conflicts of Interest

The authors declare no conflicts of interest.

## Supporting information


**Figure S1:** The association between expression level of AGTRAP and clinical features.
**Figure S2:** The relationship between the expression of AGTRAP in glioma and prognosis.
**Figure S3:** The expression distribution of AGTRAP in glioma tissues in the single‐cell RNA sequencing datasets.
**Figure S4:** (A) Flow cytometry was used to detect the apoptosis of A172 cells after AGTRAP knockdown.
**Figure S5:** (A, B) The level of IL‐6 was quantified by qRT–PCR (A) and ELISA (B) in glioma cells.
**Figure S6:** (A, B) Western blot analysis of p‐NF‐κB P65/NF‐κB P65 in glioma cells.
**Figure S7:** (A, B) Glioma cells and THP‐1 cells were co‐cultured using the Transwell system to evaluate glioma cell‐driven THP‐1 cell migration.
**Figure S8:** The genome characteristics between high and low AGTRAP groups.

## Data Availability

Data supporting the findings of this study are available upon reasonable request from the corresponding authors.
