# Supplementary material for: Angiotensin II Receptor‐Associated Protein (AGTRAP) Enhances Glioma Cell Survival Through the IL‐6/JAK2/STAT3 Pathway and Correlates With an Immunosuppressive Microenvironment
Source: CNS Neurosci Ther. 2026 Feb 13;32(2):e70796. doi: 10.1002/cns.70796 (PMC12905009; doi:10.1002/cns.70796)
Supplement: Supplementary file 1 — Figure S1: The association between expression level of AGTRAP and clinical features. Figure S2: The relationship between the expression of AGTRAP in glioma and prognosis. Figure S3: The expression distribution of AGTRAP in glioma tissues in the single‐cell RNA sequencing datasets. Figure S4: (A) Flow cytometry was used to detect the apoptosis of A172 cells after AGTRAP knockdown. Figure S5: (A, B) The level of IL‐6 was quantified by qRT–PCR (A) and ELISA (B) in glioma cells. Figure S6: (A, B) Western blot analysis of p‐NF‐κB P65/NF‐κB P65 in glioma cells. Figure S7: (A, B) Glioma cells and THP‐1 cells were co‐cultured using the Transwell system to evaluate glioma cell‐driven THP‐1 cell migration. Figure S8: The genome characteristics between high and low AGTRAP groups. [file CNS-32-e70796-s001.docx]

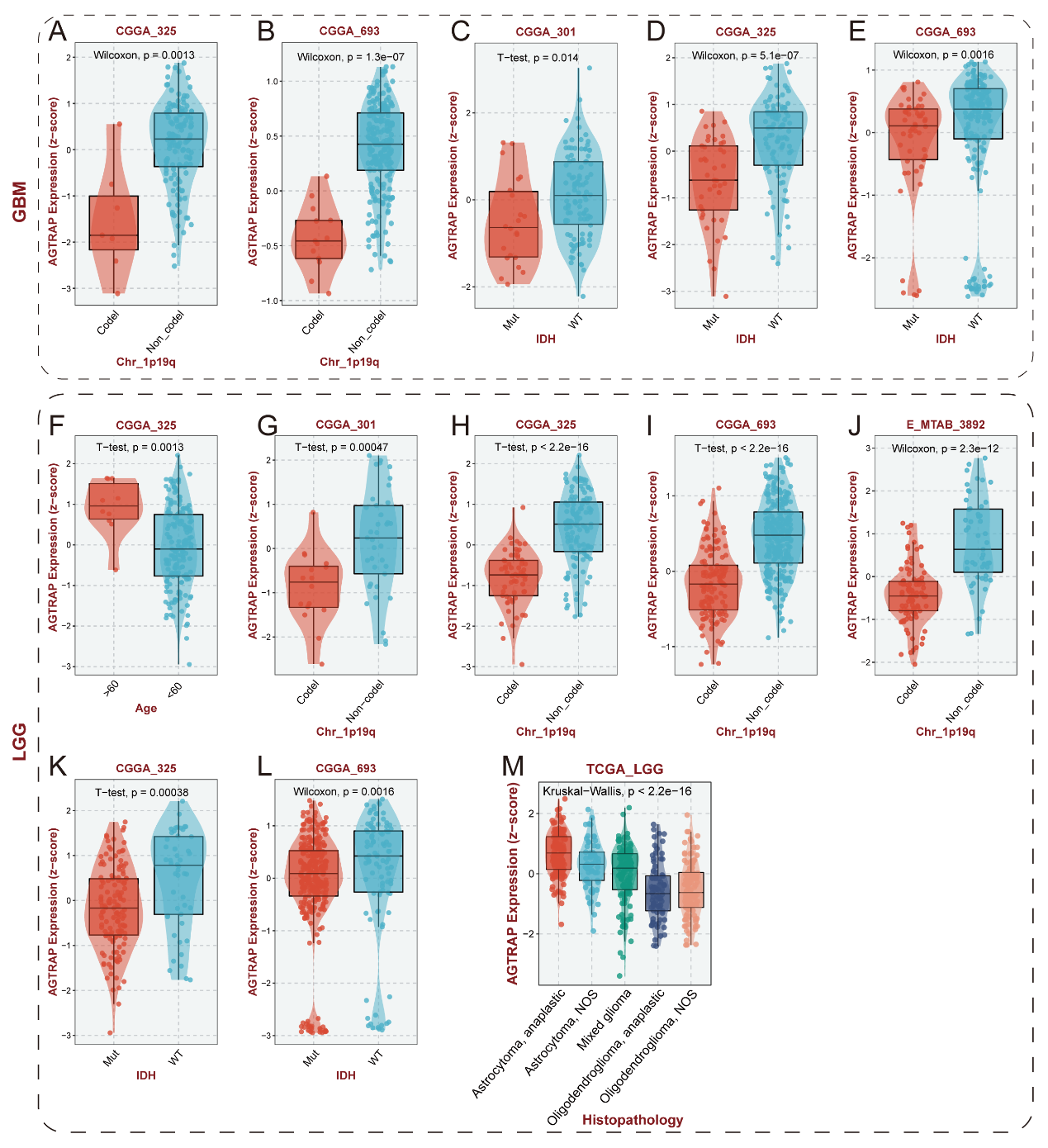


Figure S1. The association between expression level of AGTRAP and clinical features

(A-E) The different expression levels of AGTRAP in different 1p19q status (A-B) and IDH status (C-E) in GBM cohorts. (F-M) The different expression levels of AGTRAP in different age (F), 1p19q status (G-J), IDH status (K-L), and histopathologies (M) in LGG cohorts.


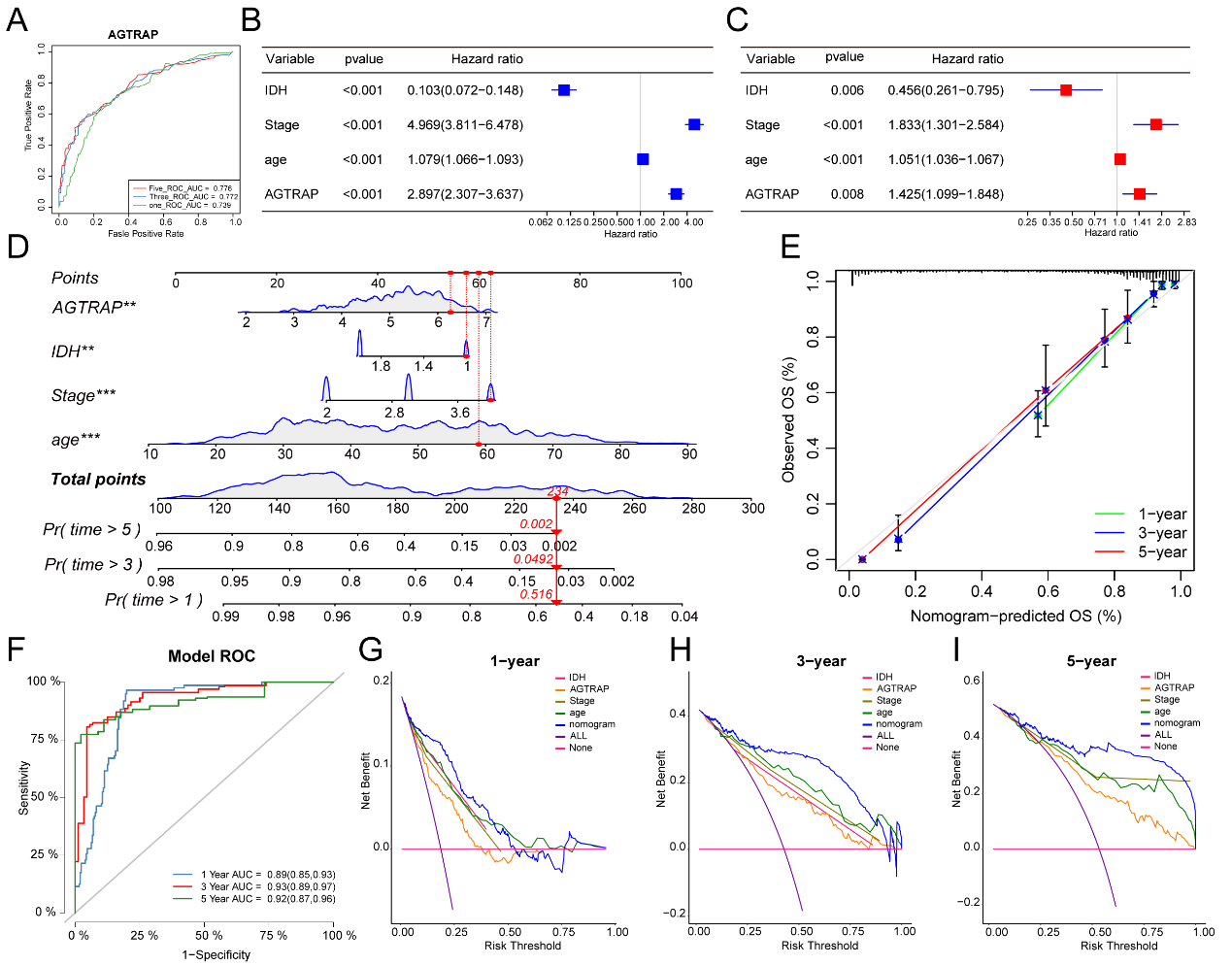


Figure S2. The relationship between the expression of AGTRAP in glioma and prognosis

(A) The ROC curve showed the prediction ability of AGTRAP in 1, 3, and 5 years. (B-C) The univariate (B) and multivariate (C) COX analyses of IDH status, WHO stages, age, and AGTRAP. (D) The nomogram of AGTRAP and other clinical features in TCGA cohorts. (E) Prediction of the calibration curve of the line graph. (F) The ROC curve of nomogram model. (G-I) The decision analysis showed the clinical efficiency of the AGTRAP in TCGA cohort at 1, 3, and 5 years. The nomogram model exhibited the highest net benefit stably.


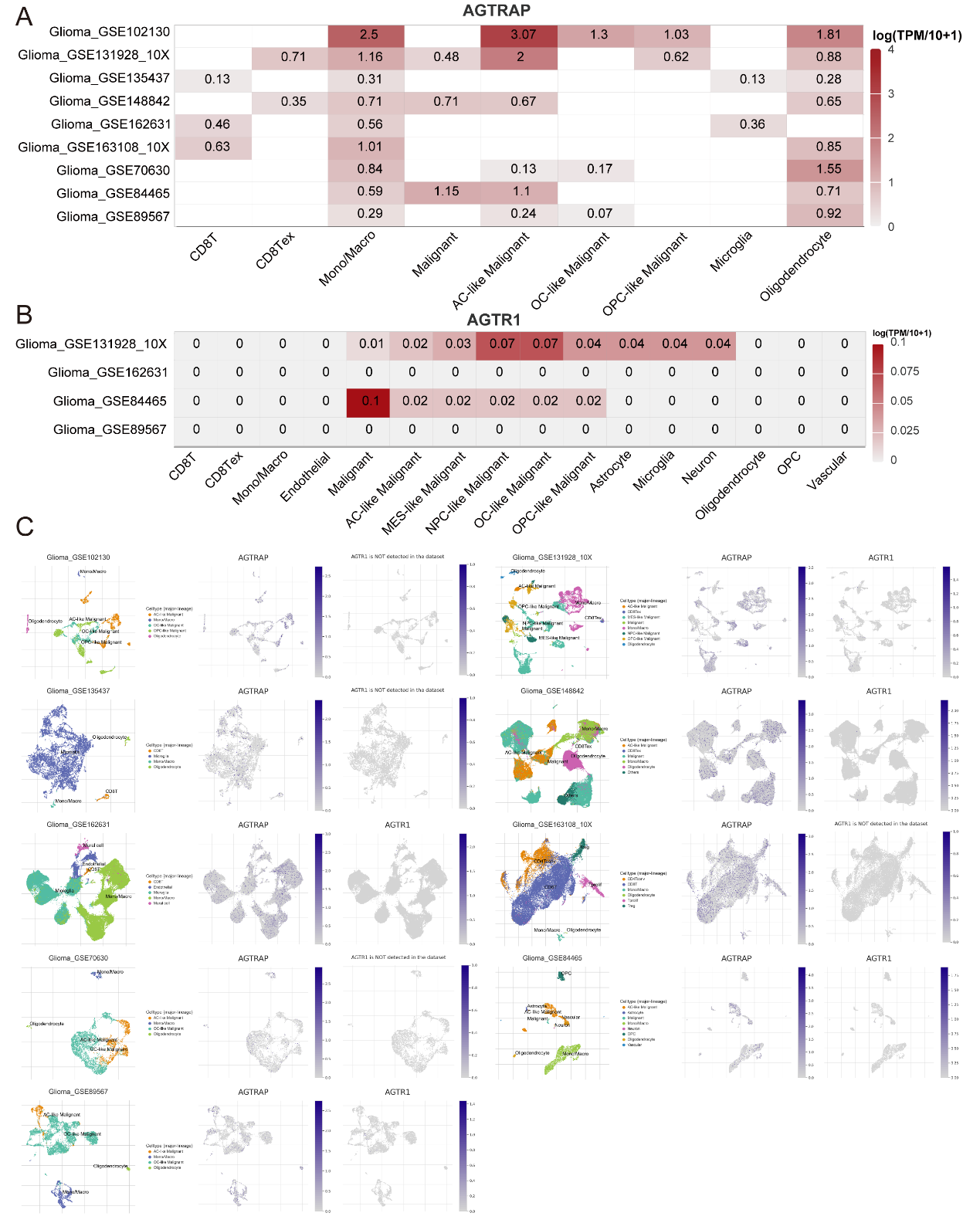


Figure S3. The expression distribution of AGTRAP in glioma tissues in the single-cell RNA sequencing datasets

(A, B) The heatmap showed AGTRAP and AT1R (AGTR1) expression in different glioma single-cell RNA sequencing datasets. (C) the UMAP plots illustrated the expression of CDCA2 in different cell types based on the GSE102130, GSE131928, GSE135437, GSE148842, GSE162631, GSE163108, GSE70630, GSE84465, and GSE89567.


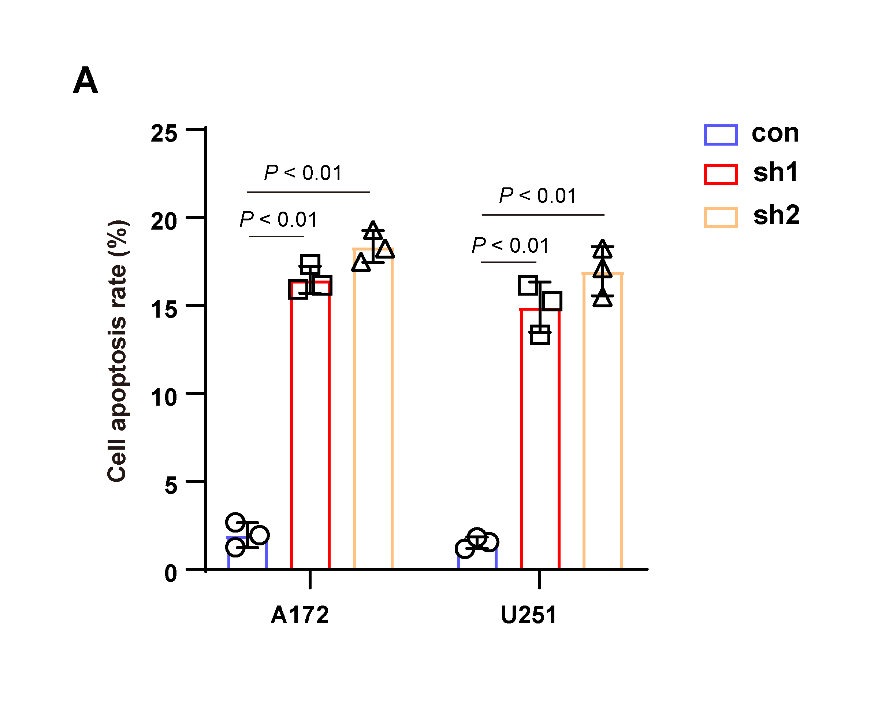


Figure S4. (A) Flow cytometry was used to detect the apoptosis of A172 cells after AGTRAP knockdown.


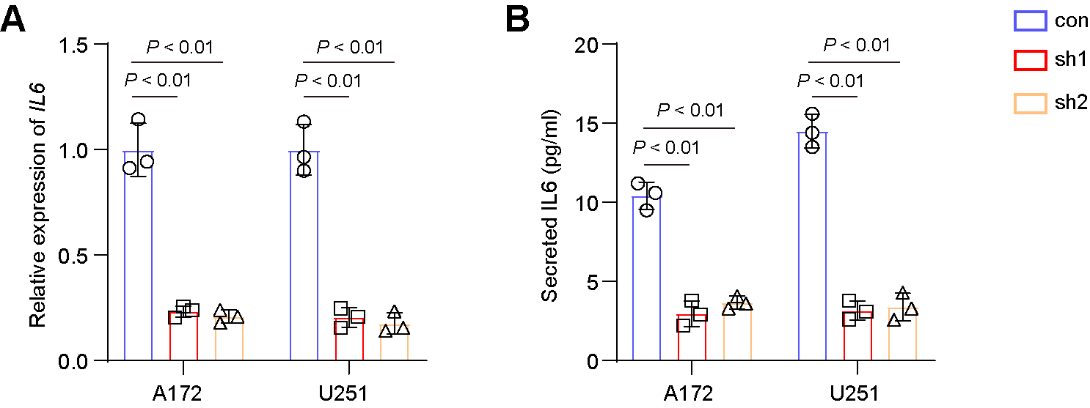


Figure S5. (A, B) The level of IL-6 was quantified by qRT–PCR (A) and ELISA (B) in glioma cells.


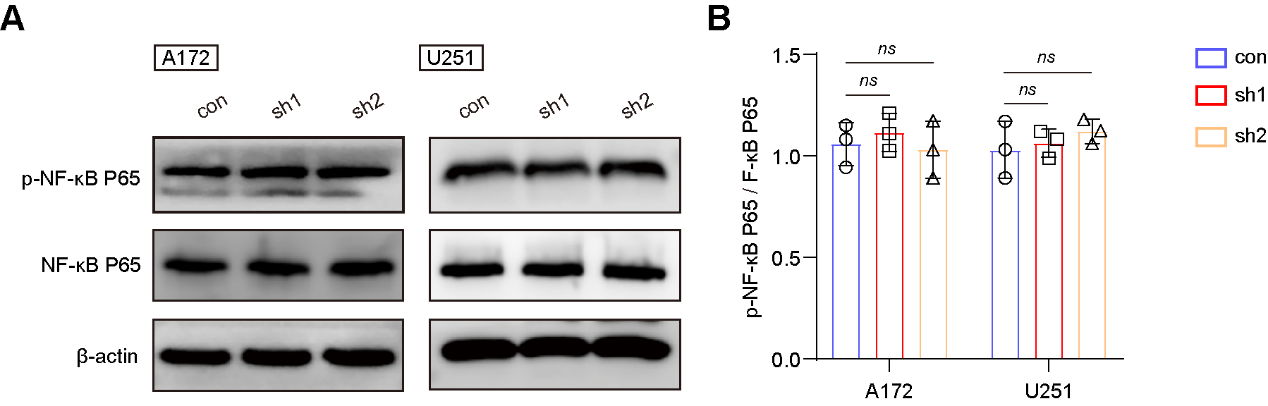


Figure S6. (A, B) Western blot analysis of p-NF-κB P65/ NF-κB P65 in glioma cells.


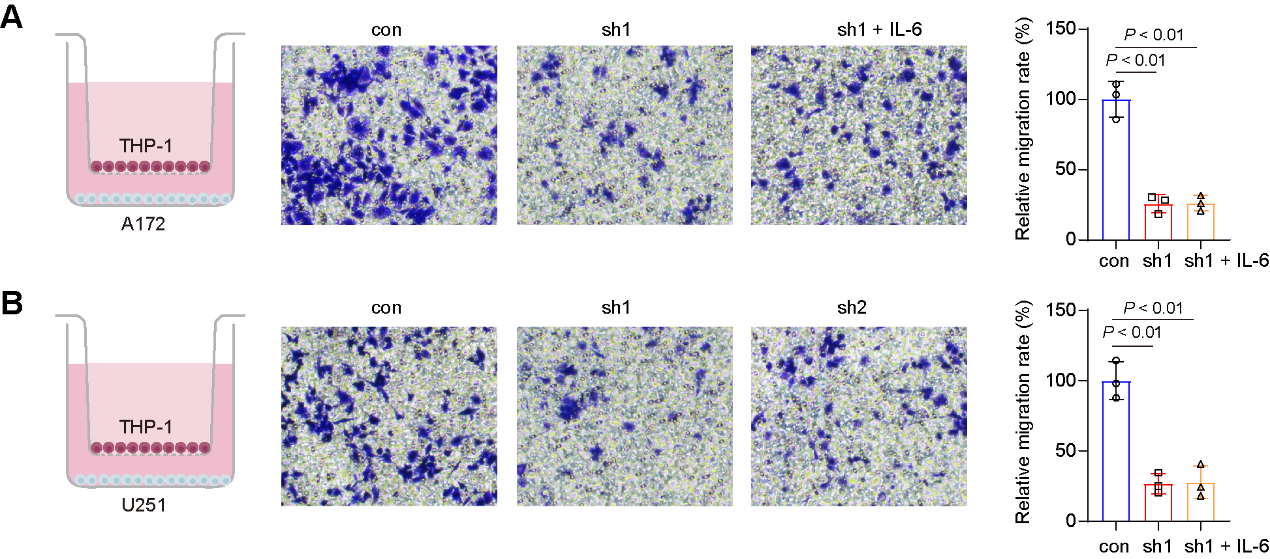


Figure S7. (A, B) Glioma cells and THP-1 cells were co-cultured using the Transwell system to evaluate glioma cell-driven THP-1 cell migration.


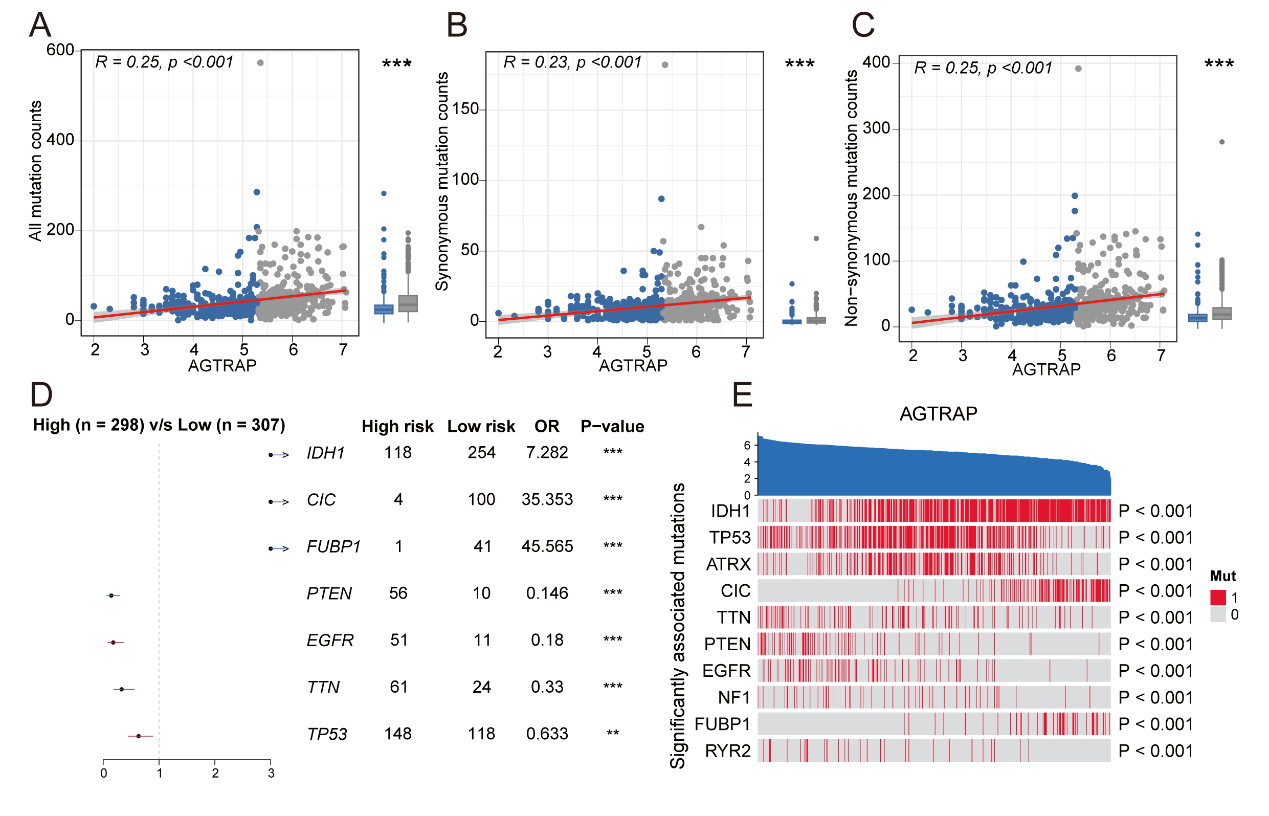


**Figure S8. The genome characteristics between high and low AGTRAP groups**

(A-C) The relationship between all mutation (A), synonymous (B) and non-synonymous (C) counts and the AGTRAP levels, respectively. (D) Forest maps show differences of glioma patients in gene mutations in the high and low AGTRAP groups. (E) Permutation test showed the relationship between top 10 mutations in gliomas and AGTRAP.
